# Informatics framework of traditional Sino-Japanese medicine (Kampo) unveiled by factor analysis

**DOI:** 10.1007/s11418-015-0946-0

**Published:** 2015-10-26

**Authors:** Taketo Okada, Farit Mochamad Afendi, Mami Yamazaki, Kaori Nakahashi Chida, Makoto Suzuki, Rika Kawai, Miyuki Kim, Takao Namiki, Shigehiko Kanaya, Kazuki Saito

**Affiliations:** Faculty of Pharmaceutical Sciences at Kagawa Campus, Tokushima Bunri University, 1314-1 Shido, Sanuki, Kagawa 769-2193 Japan; Graduate School of Information Science, Nara Institute of Science and Technology, 8916-5 Takayama-cho, Ikoma, Nara 630-0192 Japan; Department of Statistics, Bogor Agriculture University, Gedung Fateta, Kampus IPB Darmaga, PO Box 220, Bogor, 16680 Indonesia; Graduate School of Pharmaceutical Sciences, Chiba University, 1-8-1 Inohana, Chuo-ku, Chiba, 260-8675 Japan; RIKEN Center for Sustainable Resource Science, 1-7-22 Suehiro-cho, Tsurumi-ku, Yokohama, 230-0045 Japan; Department of Japanese-Oriental (Kampo) Medicine, Graduate School of Medicine, Chiba University, 1-8-1 Inohana, Chuo-ku, Chiba, 260-8675 Japan

**Keywords:** Kampo, *Sho*, Factor analysis, Metabolome analysis

## Abstract

Kampo, an empirically validated system of traditional Sino-Japanese medicine, aims to treat patients holistically. This is in contrast to modern medicine, which focuses in principle on treating the affected parts of the body of the patient. Kampo medicines formulated as combinations of crude drugs are prescribed based on a Kampo-specific diagnosis called *Sho* (in Japanese), defined as the holistic condition of each patient. Therefore, the medication system is very complex and is not well understood from a modern scientific perspective. Here, we show the informatics framework of Kampo medication by multivariate factor analysis of the elements constituting Kampo medication. First, the variation of Kampo formulas projected by principal component analysis (PCA) indicated that the combination patterns of crude drugs were highly correlated with *Sho* diagnoses of Deficiency and Excess. In an opposite way, partial least squares projection to latent structures (PLS) regression analysis could also predict Deficiency/Excess only from the composed crude drugs. Secondly, to chemically verify the correlation between Deficiency/Excess and crude drugs, we performed mass spectrometry (MS)-based metabolome analysis of Kampo prescriptions. PCA and PLS regression analysis of the metabolome data also suggested that Deficiency/Excess could be theoretically explained based on the variation in chemical fingerprints of Kampo medicines. Our results show that factor analysis of Kampo concepts and of the metabolomes of Kampo medicines enables interpretation of the complex system of Kampo. This study will theoretically form the basis for establishing traditionally and empirically based medications worldwide, leading to systematically personalized medicine.

**Electronic supplementary material:**

The online version of this article (doi:10.1007/s11418-015-0946-0) contains supplementary material, which is available to authorized users.

## Introduction

With a history dating back 1,600 years, Kampo is a traditional Japanese medicine of therapeutic strategies and diagnostics adapted from traditional Chinese medicine (TCM). Kampo medicines are prescribed and generally prepared with combinations of crude drugs derived from herbs, animals, and minerals. Curative effects are based on synergism between pharmacologically and biologically active constituents producing minimum side-effects. Currently, 254 regulated crude drugs, as well as their processed forms, and 22 Kampo medicines are listed in the Japanese Pharmacopoeia, 16th edition [1], and 294 over-the-counter prescriptions were approved in 2012. A revival of Kampo has been accompanied by scientific re-evaluation of its relevance to modern healthcare [2, 3].

**Fig. 1:**
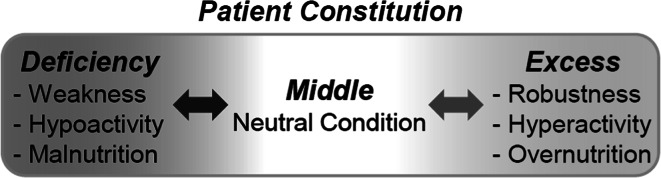
Patient constitutions according to Kampo diagnostic criteria *Sho*. Relationships between patient conditions of Deficiency, Middle, and Excess

Kampo is a personalized holistic treatment system for improving the disease states of a patient by returning the patient to a balanced state. The Japanese term *Sho* refers to the fundamental diagnosis of the patient’s conditions and symptoms and is the term given to the summarization of the diagnostic process [4–6]. A patient’s constitution is diagnosed based on the three states in *Sho*—Deficiency (*Kyo*), Middle (*Kang*), and Excess (*Jitsu*), referring to states of weakness/hypoactivity/malnutrition, neutrality, and robustness/hyperactivity/overnutrition, respectively (Fig. 1). This systemic diagnostic method has been empirically established based on clinical and pharmacological evidence accumulated over many years. The method is quite unique to Kampo and differs from the diagnostic approach in contemporary medicine, which treats abnormal patient conditions by focusing on individual conditions and symptoms. Accordingly, the prescriptions of Kampo medicines are not limited to the treatment of only targeted symptomatic disease.

Recently we proposed that informatics based on the comprehensive and simultaneous analysis of numerous factors may be clarified to classify the complex relationships among crude drugs and formulas, chemical constituents, and pharmacological and biological effects in traditional and modern medicine (reviewed in Refs. [7–9] ). In the present study, we investigated the relationship between the diagnostic criteria *Sho* and the prescriptions of Kampo medicines based on statistical factorial analysis. Specifically, this study focused on the complex correlation between the combination of patterns of crude drugs in formulating Kampo medicines and a *Sho* diagnosis of Deficiency/Excess. To systematically understand and interpret the theory of empirical medication in Kampo, multivariate statistics including principal component analysis (PCA) and partial least squares projection to latent structures (PLS) modeling were applied to the correlation between crude drug patterns and Deficiency/Excess. Metabolome analysis, a comprehensive and global chemical analysis of metabolites contained in samples of decoctions of Kampo prescriptions actually prepared from mixtures of crude drugs was also incorporated using mass spectrometry (MS), to substantiate the relationships between Kampo formulas and Deficiency/Excess using the chemical fingerprints of Kampo prescriptions based on the similarities and differences among their chemically complex features. In this study, we begin to unveil the complex system of Kampo medication.

## Materials and methods

### Kampo formulas

Kampo formulas analyzed in this study are listed in the KAMPO section of the KNApSAcK family database [8, 10] and in several Kampo reference texts [11–25]. Medicinal resources of crude drugs used in Kampo formulas are listed in two references texts for crude drugs [1, 26] and are described along with their scientific names and medicinally active region in Table S1. Kampo formulas are introduced using the structured Romanized notation recommended by The Japan Society of Oriental Medicine (Tokyo, Japan), and are abbreviated in subsequent appearances. For metabolome analysis, 25 Kampo prescriptions containing Cinnamon bark (Cinnamomi Cortex), as well as 9 other prescriptions, were selected from Refs [11, 13].

### Preparation of decoctions of Kampo prescriptions for metabolome analysis

Crude drugs for the preparation of 34 Kampo prescriptions (Table S2) were purchased from Uchida Wakanyaku (Tokyo, Japan) and Tsumura (Tokyo, Japan). The decoctions for metabolome analysis were prepared in accordance with the standard method clinically used at the Diagnosis and Treatment Department of Kampo Medicine, Chiba University Hospital (Chiba, Japan) as follows. Kampo prescriptions tested were packed in an L-size filter bag (Uchida Wakanyaku). The packed prescription was boiled with 600 mL of water for 60 min by using a decocting pot with an electric heater (HMJ3-1000 W; Uchida Wakanyaku).

### Acquisition of chemical fingerprints by MS-based metabolome analysis of Kampo prescriptions

High-accuracy quadrupole time-of-flight (Q-TOF)–MS analysis by direct infusion was performed for acquisition of the chemical fingerprints of Kampo prescriptions using a Q-TOF mass spectrometer (Q-TOF micro™ Mass Spectrometer; JASCO International, Tokyo, Japan) and the resolution was set at 5,000. Ionization of the analyzed samples was performed by positive electrospray ionization (ESI) at *m*/*z* range of 85–1,200. The flow rates of cone and desolvation N_2_ gases were set at 50 and 500 L h^−1^, respectively. In the positive ESI source, capillary and sample cone voltages were set at 2,800 and 30 V, respectively, with the desolvation and source temperatures set to 150 and 100 °C, respectively. The ion energy was set at 2.0 V in the *Q* setting. TOF flight tube and tube lens voltages were set at 5,630 and 90 V, respectively, and a microchannel plate detector was set at 2,400 V.

The spectral intensity in MS analysis was acquired under the unsaturated condition of peak detection. Test samples were diluted 100-fold with water and injected by syringe pump at a constant rate. The basal conditions of Q-TOF–MS analysis were calibrated by measured values of sodium formate solution (0.1 % formic acid and 5 mM NaOH/90 % acetonitrile). MS data were collected at a scanning rate of 1.0 s per one data scan with 0.1 s of internal delay. In addition, leucine-enkephalin, of which the *m*/*z* value in positive ESI analysis was 556.277 ([M + H]^+^), was analyzed as a reference compound for calibration of the measured values using a syringe pump with a lock spray module, permitting one data scan every 5.0 s.

### Processing of metabolome data for multivariate analysis

A metabolomics data matrix for multivariate analysis was constructed by *m/z* values and peak intensities of mass spectra (Table S3). Data points of *m*/*z* values were reduced by integration of the peak intensities per *m*/*z* range of 0.5 (*m*/*z*: $85.0,85.5,86.0 \ldots 1199.0,1199.5$). Accordingly, a data matrix consisting of 2,230 *m*/*z* values and the corresponding peak intensities was generated. The acquired metabolome data were applied to PCA and PLS regression analysis.

### PCA

Kampo medicines are blended herbal formulas composed of several crude drugs sourced from plants, animals, and minerals that are thought to possess complex interactions. The *i*th Kampo formula can be represented by a vector consisting of quantities of each crude drug in the composition, that is, ***x***_*i*_ [= ($x_{i1} ,x_{i2} , \ldots ,x_{ij} , \ldots ,x_{iM}$)]. Here, *x*_*ij*_ represents the quantity of *j*th crude drug in *i*th formulas. Thus, the crude drugs in all formulas examined were represented by a matrix as in Eq. (1).

1 $$\,X = \left( {\begin{array}{*{20}c} {x_{11} } & {x_{12} } & \ldots & {x_{1M} } \\ {x_{21} } & {x_{21} } & \ldots & {x_{2M} } \\ \ldots & \ldots & \ldots & \ldots \\ {x_{N1} } & {x_{N2} } & \ldots & {x_{NM} } \\ \end{array} } \right)$$

Here, total numbers of Kampo formulas and composite crude drugs are denoted by *N* and *M*, respectively. PCA was applied to the matrix to extract the independent factors [27]. The *k*th principal component is a linear combination of the principal coefficients *b*_*kj*_, that is, $\,Z_{k} = b_{k1} X_{1} + b_{k2} X_{2} + \cdots + b_{kM} X_{M}$. Here, *b*_*k*1_ was normalized as unity ($\,\sum\nolimits_{j = 1}^{M} {b_{kj}^{2} } = 1$). Variables and elements are denoted with upper and lower case letters, respectively. Correlation between the principal components *Z*_*k*_ and $Z_{k'}$($k \ne k'$) was zero, and the first PC (*Z*_1_) has the largest variance, the second PC was that with the second-largest variance, etc. Two parameters, proportion and factor loadings, are used to interpret the distribution of PCA. Proportion Pr(*Z*_*k*_) is represented by the ratio of variance of the *k*th PC score Var(*Z*_*k*_) to the total variance, and factor loading *r*(*Z*_*k*_, *X*_*j*_) is represented by the correlation coefficient between the *k*th PC score and the *j*th variable.

### PLS regression analysis

Kampo formulas were analyzed by reciprocal attributes Deficiency and Excess in *Sho* based on PLS regression analysis. If the *i*th formula has the attribute Deficiency, then *y*_*i*_ was set to −1, and if it has the attribute Excess, then *y*_*i*_ was set to 1. The discrimination function is expressed by Eq. (2).

2 $$y = b_{0} + b_{1} X_{1} + \cdots + b_{j} X_{j} + \cdots + b_{M} X_{M}$$

Here, $b_{0} ,b_{1} , \ldots ,b_{M}$ are regression coefficients. A multiple linear regression model is an effective method when the number of variables *M* is much smaller than that of samples *N*, but it should be noted that in the case of datasets with strong collinearity between variables, regression coefficients cannot reflect the relationship between variables **X** with the effect **y**. In other words, two variables *X*_*u*_ and *X*_*v*_ are positively correlated to *y* but regression coefficients *b*_*u*_ and *b*_*v*_ are not always positive, which leads to difficulty in interpreting the relationship between *X*_*u*_/*X*_*v*_ and *y*. A multivariate linear equation expressing *y* with variables derived using PCA by transforming the original variables can avoid the problem of collinearity between variables, but interpretation of the model equation becomes complicated because, *Z*_*k*_ is a combination of the original variables *X*_*j*_ ($j = 1,2, \ldots ,M$). A PLS model is a method for linearly relating the data matrix **X(**consisting of *N* × *M*) to vector **y** = (consisting of *N* × 1), as represented by Eqs. (4) and (5). This makes it possible to overcome the two aforementioned issues, collinearity between variables and indirect interpretation [7].

3 $${\mathbf{y}} = {\bar{\mathbf{y}}} + \sum\limits_{k = 1}^{A} {{\mathbf{t}}_{k} } q_{k} + {\mathbf{e}}$$

4 $${\mathbf{X}} = {\bar{\mathbf{X}}} + \sum\limits_{k = 1}^{A} {{\mathbf{t}}_{k} } {\mathbf{p}}_{k}^{\text{T}} + {\mathbf{E}}$$

Here **p**_*k*_ and *q*_*k*_ are the loading vectors of **X**, and the coefficient of **y** for the *k*th component, respectively. *A* is the number of components and **t**_*k*_ is a score vector for the *k*th component. **E** and **e** represent the residual matrix and vector, respectively. The number of components, *A*, is determined by maximization of *Q*^2^ by Eq. (5).

5 $$Q^{2} = 1 - \frac{{\sum {(y_{\text{obs}} - y_{\text{pred}} )^{2} } }}{{\sum {y_{\text{obs}}^{2} } }}$$

*A* with *Q*^2^, a value closest to 1 is the best linear regression equation to explain the relation of **X** to effect **y**. The multivariate linear relation denoted by Eq. (2) can be easily derived from Eqs. (3) and (4).

## Results and discussion

### Factorial analysis of Kampo formulas

**Fig. 2:**
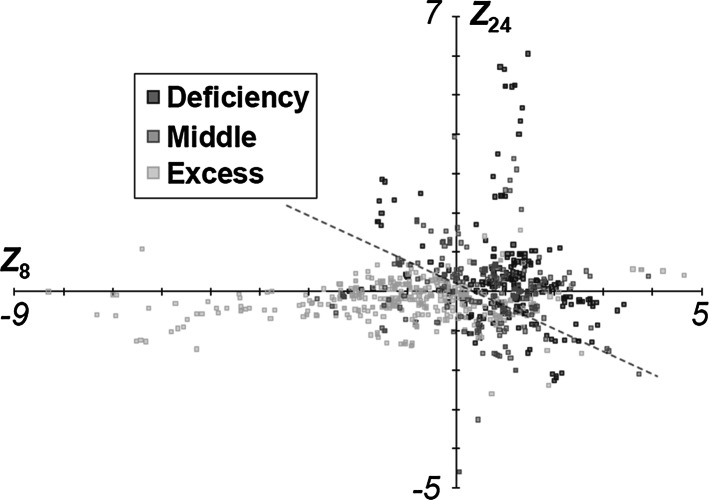
PCA projections for Kampo formulas based on *Sho* patient constitutions. Classification of Kampo medicines prescribed for Deficiency, Middle, and Excess by PC 8 and 24

PCA of unsupervised learning and PLS modeling of supervised learning are very popular in multivariate analysis and have been applied in previous studies to analyze ingredients and metabolome of crude drugs (reviewed in Refs. [7, 28] ). Prior to this study, a comprehensive classification of Kampo formulas (*N* = 826) was performed using PCA (Fig. 2). For this multivariate analysis, a matrix represented in Eq. (1) was set using the quantities of crude drugs (*M* = 159) composing each Kampo formula. In the present analysis, PC 1–26 axes where each PC axis accounts for >1 % of variance were selected, and their total variance was 77.9 %. We examined *p* values by *t* test in differences in *Sho* diagnoses of Deficiency and Excess. Of 26 PCs, PC 8 (*p* = 9.2 × 10^−37^) and PC 24 (*p* = 4.9 × 10^−17^) were the most significantly different (Fig. S1); thus, the variation of 826 Kampo formulas was analyzed in a two-dimensional space using PC 8 and PC 24.

The scores of PCA assigned to Kampo formulas prescribed for Deficiency (382 formulas), Middle (218 formulas), and Excess (226 formulas), were projected onto a two-dimensional space (Fig. 2). In this unsupervised analysis, a discriminant boundary between the formulas prescribed for Deficiency and Excess was broadly observed in both axes (dotted line in Fig. 2). The formulas prescribed for Middle also formed a cluster at a nearby discriminant boundary between the formulas for Deficiency and Excess. The contribution of crude drugs for variation of formulas was indicated by the factor loading in PCA. For example, Pueraria root (Puerariae Radix) (0.673) and apricot kernel (Armeniacae Semen) (−0.293) in PC 24, and fennel (Foeniculi Fructus) (0.041) and ass-hide glue (Asini Gelatinum) (−0.252) in PC 8 contributed to the variation of formulas in each degree. These results suggested that the prescribing theory of Kampo medicines may be considered from the point of view of the formulations and quantities of the crude drugs composing the medicines.

**Fig. 3:**
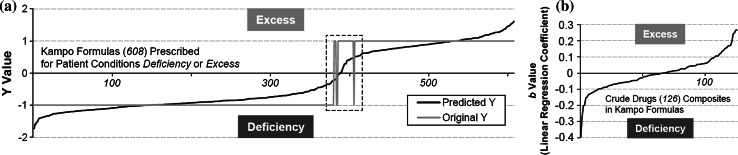
Classification of Kampo formulas based on *Sho* patient constitutions by PLS regression analysis. **a** *Y* values assigned to Kampo formulas. Negative and positive *Y* values suggest Deficiency and Excess, respectively. *Black line* indicates predicted *Y* values of Kampo formulas. *Gray line* shows the actual *Y* values, where −1 and 1 indicate Deficiency and Excess, respectively. The 7 formulas surrounded by a *dotted line* were contrary to prescriptions for Deficiency and Excess. **b** Regression coefficients (*b*) of crude drugs in Kampo medicines. Negative and positive values suggest the contribution of crude drugs to Deficiency and Excess, respectively

To verify the results of PCA, the difference of quantities in the composition of crude drugs between the prescriptions for Deficiency and Excess was examined using PLS regression analysis. From the quantities of composite crude drugs (*M* = 126) as well as PCA, the distinctions between Kampo formulas (*N* = 608) assigned to Deficiency (382 formulas) and Excess (226 formulas) were predicted based on a PLS model (Fig. 3). The regression model was constructed using 12 axes, because the *Q*^2^ value, which is estimated by cross-validation in PLS modeling, indicated that the fractions of total variation among variables *X* or *Y* was maximized in this case (0.863). The distinctions between 601 of 608 Kampo formulas (98.8 %) were accurately predicted from the combination patterns of crude drugs (Fig. 3a).

Figure 3b shows the correlation between the linear regression coefficients, which are the *b* values of the linear equation predicted in Eq. (2), and the use rates of 126 crude drugs used in Kampo formulas prescribed for Deficiency or Excess by PLS regression analysis. The contribution of *j*th crude drug prescribed for Deficiency or Excess can be easily assessed by the coefficient *b*_*j*_; if *b*_*j*_ of *j*th crude drug is positive, it is used more frequently for Excess, while negative *b*_*j*_ values show that the use rate of *j*th crude drug is greater for Deficiency. Crude drugs that were assigned coefficient *b* less than −0.1 and >0.1 were selected in Table 1. For example, Japanese angelica root (Angelicae Radix), which is used in 179 of the 608 formulas (29.4 %) was predicted to contribute in cases of Deficiency (*b* = −0.153). Japanese angelica root generally works to replenish blood via a warming action, resulting in the improvement of weak constitution. This pharmacological role well agrees with its prescription in Kampo formulas for Deficiency. Conversely, Scutellaria root (Scutellariae Radix), which is used in 148 of 608 formulas (24.3 %) was predicted to contribute in cases of Excess (*b* = 0.267). Scutellaria root generally removes and brings down high fever, and this pharmacological role also agrees with its application in Kampo formulas prescribed for Excess. In addition to Scutellaria root, Bupleurum root (Bupleuri Radix), used in 104 formulas (17.1 %) also has antipyretic action, and was predicted to contribute in cases of Excess (*b* = 0.158). In actual practice, Kampo medicines composed of both Bupleurum root and Scutellaria root are often prescribed for Excess. In this study, 58 of 608 formulas contained both Bupleurum root and Scutellaria root, and 52 of these 58 formulas are generally prescribed for Excess. Coptis rhizome (Coptidis Rhizoma) is also often used with Scutellaria root in Kampo formulas (57 of the 608 formulas in this study), and these crude drugs resolve excessive fever in the body by their joint action. Interestingly, Coptis rhizome was predicted to contribute in cases of Deficiency (*b* = −0.118) in contrast to the prediction for Scutellaria root. This result is consistent with the actual use of Kampo medicines containing both Scutellaria root and Coptis rhizome, as 20 and 37 formulas of the total 57 formulas are separately prescribed for Deficiency and Excess, respectively. Thus, PLS regression analysis predicted almost the same patterns of use for Deficiency and Excess as those in practice, suggesting that Deficiency and Excess can be interpreted by the composition of crude drugs.

**Table 1:** Linear regression coefficients *b* of PLS regression analysis assigned to crude drugs composing Kampo formulas prescribed for Deficiency and Excess

Crude drug	*b* Value (<−0.1)	No. of formulas	Percentage
Trichosanthes root	−0.397	6	0.99
Oyster shell	−0.276	17	2.80
Bamboo shavings	−0.202	20	3.29
Processed ginger	−0.200	86	14.14
Japanese angelica root	−0.153	179	29.44
Hemp fruit	−0.149	20	3.29
Rehmannia root (steamed)	−0.134	19	3.13
Lycium bark	−0.130	6	0.99
Immature orange	−0.127	66	10.86
Orange	−0.123	12	1.97
Coptis rhizome	−0.118	66	10.86
Achyranthes root	−0.113	14	2.30
Glycyrrhiza	−0.109	429	70.56
Euodia fruit	−0.106	21	3.45

Crude drug	*b* Value (>0.1)	No. of formulas	Percentage
Scutellaria root	0.267	148	24.34
Akebia stem	0.265	19	3.13
Rhubarb	0.249	97	15.95
Polyporus sclerotium	0.243	17	2.80
Common rush	0.197	13	2.14
Trichosanthes seed	0.171	8	1.32
Ephedra herb	0.170	47	7.73
Areca peel	0.169	13	2.14
Bupleurum root	0.158	104	17.11
Saussurea root	0.155	37	6.09
Saposhnikovia root and rhizome	0.148	41	6.74
Aralia rhizome	0.138	10	1.64
Japanese cherry bark	0.134	8	1.32
Plantago seed	0.121	15	2.47
Pueraria root	0.119	17	2.80
Cyperus rhizome	0.107	37	6.09
Schisandra fruit	0.106	26	4.28
Magnolia bark	0.105	59	9.70

The *b* values less than −0.1 and >0.1 were listed

As shown by the dotted line in Fig. 3a, *Y* values −0.221 to 0.479 predicted for 3 of 5 formulas—Keishi-ka-shakuyaku-daio-To (KSTSD; Keishikashakuyakudaioto) variants, Daio-bushi-To (DBST; Daiobushito) and Mao-bushi-saishin-To (MBST; Maobushisaishinto)—were contrary to the prescriptions for Deficiency and Excess. KSTSD was predicted for Deficiency but is generally prescribed for Excess. The formula is prepared from Keishi-ka-shakuyaku-To (KSTS; Keishikashakuyakuto), usually prescribed for Deficiency, by adding rhubarb (Rhei Rhizoma) (*b* = 0.249 in Fig. 3b), which is often added to Excess prescriptions (see nos. 6 and 7 in Tables 2 and S2). This difference of use from Excess to Deficiency may be due to its use as a combination of crude drugs in KSTS, such that the addition of rhubarb to KSTS did not affect the predicted prescription of KSTSD for Deficiency or Excess. DBST containing rhubarb is also generally prescribed for Excess, but was predicted for Deficiency, probably due to the presence of the two other crude drugs in DBST, Asiasarum root (Asiasari Radix) (*b* = −0.053) and processed aconite root (Processi Aconiti Radix) (*b* = −0.099), because they are used to treat Deficiency. MBST was predicted for Excess but prescribed for Deficiency, and also contains Asiasarum root and processed aconite root, which play important roles in Deficiency; however, the effect of Ephedra herb (Ephedrae Herba) (*b* = 0.170 in Fig. 3b) for Excess may have contributed to this difference.

**Table 2:** 34 Kampo prescriptions analyzed by direct infusion Q-TOF–MS for metabolomic analysis

Prescription no.	Kampo prescription	Patient constitution according to *Sho*
1	Keishi-To	Deficiency
2	Keishi-ka-ogi-To	Deficiency
3	Keishi-ka-kakkon-To	Deficiency
4	Keishi-ka-kei-To	Deficiency
5	Keishi-ka-koboku-kyonin-To	Deficiency
6	Keishi-ka-shakuyaku-To (KSTS)	Deficiency
7	Keishi-ka-shakuyaku-daio-To (KSTSD)	Excess
8	Keishi-ka-shakyaku-shokyo-ninjin-To	Deficiency
9	Keishi-ka-ryukotsu-borei-To	Deficiency
10	Keishi-ni-eppi-Itto (Keishinieppiitto)	Excess
11	Keishi-ni-mao-Itto (Keishinimaoitto)	Deficiency
12	Keishi-mao-kakuhan-To	Middle
13	I-rei-To	Middle
14	Kakkon-To	Excess
15	Kakkon-To-ka-senkyu-shin’i	Excess
16	Kakkon-ka-hange-To	Excess
17	Goshaku-San	Deficiency
18	Saiko-keishi-To	Deficiency
19	Keishi-kanzo-To (Keishikanzoto)	Deficiency
20	Keishi-kyo-kei-ka-bukuryo-To (Keishikyokeikabukuryoto)	Deficiency
21	Keishi-kyo-shakuyaku-To (Keishikyoshakuyakuto)	Deficiency
22	Keishi-ninjin-To	Deficiency
23	Keishi-bukuryo-Gan (KBG)	Excess
24	Ogi-keishi-gomotsu-To	Deficiency
25	Ogon-To	Excess
26	Ogon-ka-hange-shokyo-To	Excess
27	Kikyo-To	Middle
28	Sai-kan-To	Excess
29	Sho-saiko-To	Excess
30	Sho-saiko-To-ka-kikyo-sekko	Excess
31	Shokyo-shashin-To	Deficiency
32	Dai-saiko-To	Excess
33	Toki-shigyaku-To	Deficiency
34	Toki-shigyaku-ka-goshuyu-shokyo-To	Deficiency

Kampo prescriptions analyzed are listed alongside the appropriate patient *Sho* diagnoses

Thus, PLS regression analysis distinguished between Kampo formulas for Deficiency and Excess as well as did PCA (Fig. 2), and enabled an assessment of the contributions of crude drugs to those groups.

### Metabolome analysis of Kampo prescriptions

**Fig. 4:**
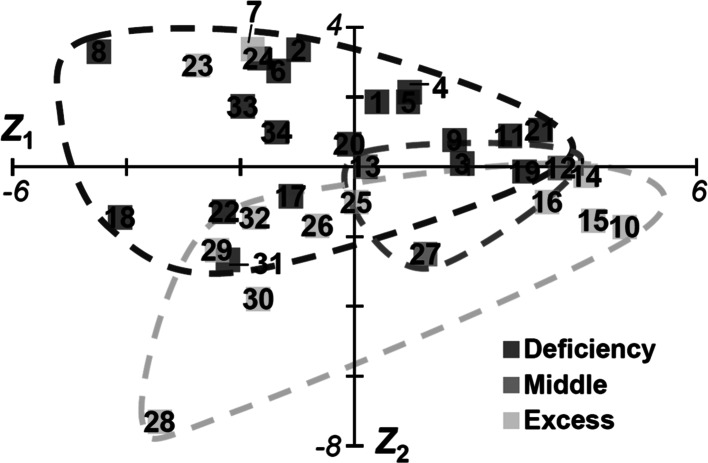
Variation of Kampo prescriptions indicated by metabolome analysis. Total variance contributions of PCs 1 and 2 were 32.5 % (*Z*_1_) and 27.7 % (*Z*_2_), respectively. The PCA result displays variation from the point of view of *Sho* diagnoses of Deficiency, Middle, and Excess

Metabolomic analysis revealed the chemical fingerprints of prescriptions to verify correlations between Kampo concepts and crude drugs. The principal crude drug, Cinnamon bark, as well as other drugs in 34 prescriptions were analyzed by Q-TOF–MS with positive ESI by direct infusion (Tables 2, S2, S3), and factor analysis was applied. Most metabolomes could be classified for *Sho* prescription by PCA (Fig. 4). Variations in prescriptions were represented by PCs 1 and 2 at 60.2 % (*Z*_1_–*Z*_2_) of variance, because PCs 1–5 contributed 89.4 % (*Z*_1_–*Z*_5_) [32.5 % (*Z*_1_) and 27.7 % (*Z*_2_), 12.2 % (*Z*_3_), 10.6 % (*Z*_4_) and 6.4 % (*Z*_5_)]. Variations in 34 prescriptions were assessed in terms of prescription for patient conditions Deficiency/Middle/Excess. All except 2 Deficiency/Excess prescriptions were classified, and Middle prescriptions were plotted around the boundary line. Compared with the results of PCA for Kampo formulas (Fig. 2), these results were perhaps due to differences in the data properties of factor analysis. Variations in prescriptions for Deficiency/Excess based on metabolomes were interpreted by lower dimensions consisting of PCs 1 and 2 (Fig. 4) rather than by combinations of PCs 8 and 24 (Fig. 2). Thus, the chemical fingerprints of Kampo prescriptions acquired by metabolomic analysis showed greater diversity than the formulation of crude drugs in Kampo medicines.

**Fig. 5:**
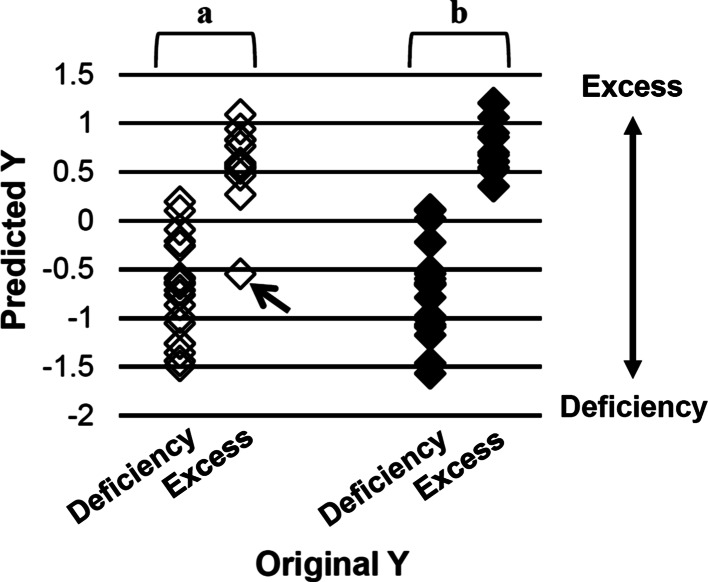
PLS regression analysis of the chemical fingerprints obtained from metabolomic analysis of Kampo prescriptions. The original *Y* values were set to ‘−1’ in Deficiency or ‘1’ in Excess. The predicted *Y* values for Deficiency or Excess were calculated from the metabolomic data of 31 Kampo prescriptions (*a*) and 30 Kampo prescriptions (when KSTSD was removed) (prescription no. 7) (*b*). *Arrow* indicates KSTSD

Metabolomic data were acquired by chemical analysis and contained latent information about crude drugs in the formulas; thus, multivariate analysis was useful in considering individual substances. KSTSD (prescription no. 7) was plotted as a Deficiency group outlier, although it is generally prescribed for Excess (Fig. 4); this may suggest that it was plotted near KSTS (prescription no. 6) on the PCA score plot because of the addition of crude drug rhubarb to KSTS. PLS regression analysis was also applied to the metabolomic data of 31 of 34 Kampo prescriptions excluding prescriptions for Middle (Fig. 5). Thus, KSTSD was predicted for Deficiency at a recognition rate of 87.1 %, but if KSTSD was removed from the 31 prescriptions, the recognition rate of the remaining 30 prescriptions for Deficiency/Excess increased to 93.3 %. The results suggest that the pharmacological and biological effects of rhubarb on Deficiency prescriptions are exceedingly high compared with other crude drugs in KSTS. Keishi-bukuryo-Gan (KBG; Keishibukuryogan) (prescription no. 23), which is usually prescribed for Excess and Middle, was plotted in the Deficiency group (Fig. 4); this outlying plot may be a result of differences in dosage form, because all samples were dissolved in water for metabolomic analysis. KBG is given in the form of a solid tablet (-*Gan* in Japanese), while the other 33 Kampo prescriptions are in the form of liquid and powder (-*To* and -*San* in Japanese, respectively). Thus, the efficacy of Kampo prescriptions appeared to differ by dosage form.

## Conclusions

In conclusion, systematic and comprehensive interpretation of Kampo medication was achieved by integration of the accumulated informatics. The factor analyses simultaneously processed a number of Kampo medicines, enabling comprehensive classification and correlation analysis of multiple factors; namely, *Sho* diagnosis of Deficiency/Excess, Kampo formulas, and the crude drugs. This finding and suggestion could be chemically substantiated by the metabolome analysis of Kampo prescriptions. The approaches in this study could lead to systems medicine, a comprehensive analysis of correlations between ingredients and practices in traditional (empirically verified) and modern medicines [7]. Factor and correlation analyses may lead to generalization of the diagnostic criteria and prescribing methods of Kampo, and be applied to other traditional medicines such as TCM and Jamu (an Indonesian traditional medicine). Moreover, Kampo medicines more suitable for the health of people in the modern age may be proposed from the new information assembled by systems medicine. Practically, TCM tends to create new combinations of crude drugs for therapeutic use, while Kampo medicine is often dependent on traditional prescriptions [29]. We believe that factor analysis of a complex medical system by informatics will have implications for systems medicine.

## Electronic supplementary material

Supplementary Table S1 (XLSX 16 kb)

Supplementary Table S2 (XLSX 14 kb)

Supplementary Table S3 (XLSX 560 kb)

Supplementary Fig. S1 (PDF 341 kb)

## Abbreviations of Kampo formulas

DBST: Daio-bushi-To (Daiobushito)
KBG: Keishi-bukuryo-Gan (Keishibukuryogan)
KSTS: Keishi-ka-shakuyaku-To (Keishikashakuyakuto)
KSTSD: Keishi-ka-shakuyaku-daio-To (Keishikashakuyakudaioto)
MBST: Mao-bushi-saishin-To (Maobushisaishinto)
