# Supplementary material for: Informatics framework of traditional Sino-Japanese medicine (Kampo) unveiled by factor analysis
Source: J Nat Med. 2015 Oct 26;70(1):107–14. doi: 10.1007/s11418-015-0946-0 (PMC4662717; doi:10.1007/s11418-015-0946-0)
Supplement: Supplementary file 4 — Supplementary Fig. S1 (PDF 341 kb) [file 11418_2015_946_MOESM4_ESM.pdf]

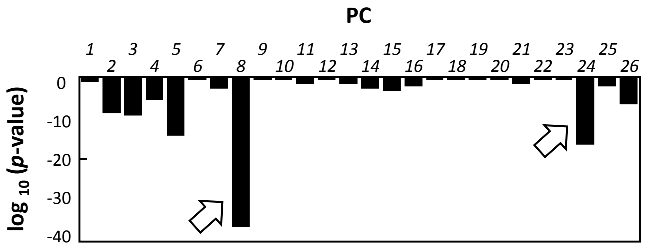

Fig. S1 Contribution of PCs for the classification of Kampo formulas in PCA projections (Fig. 2). PCs 8 and 24 had higher contributions to the classification of Kampo formulas.
